# Molybdenum Disulfide–Zinc Oxide Photocathodes for Photo-Rechargeable Zinc-Ion Batteries

**DOI:** 10.1021/acsnano.1c06372

**Published:** 2021-10-05

**Authors:** Buddha Deka Boruah, Bo Wen, Michael De Volder

**Affiliations:** † Institute for Manufacturing, Department of Engineering, University of Cambridge, Cambridge CB3 0FS, United Kingdom; ‡ Cambridge Graphene Centre, University of Cambridge, Cambridge CB3 0FA, United Kingdom

**Keywords:** zinc-ion batteries, MoS_2_/ZnO photocathodes, stacked design, photo-rechargeable batteries, photoconversion efficiency

## Abstract

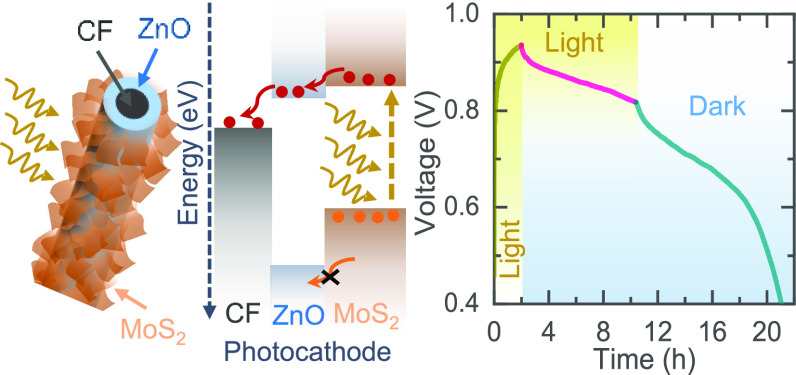

Systems for harvesting and storing solar energy have found practical applications ranging from solar farms to autonomous smart devices. Generally, these energy solutions consist of solar cells for light harvesting and rechargeable batteries to match the solar energy supply to consumption demands. Rather than having a separate energy harvesting and storing device, we report photo-rechargeable zinc-ion batteries (*h*ν-ZIBs) using a photoactive cathode composed of layer-by-layer grown zinc oxide and molybdenum disulfide. These photocathodes are capable of harvesting solar energy and storing it in the same material and alleviate the need for solar cells or power converters. The proposed photocathodes achieve photoconversion efficiencies of ∼1.8% using a 455 nm light source and ∼0.2% of solar-conversion efficiencies. Light not only allows photocharging but also enhances the battery capacity from 245 to 340 mA h g^–1^ (specific current of 100 mA g^–1^ and 12 mW cm^–2^ light intensity at 455 nm). Finally, the proposed *h*ν-ZIBs also demonstrate a capacity retention of ∼82% over 200 cycles.

Combinations of solar cells and batteries are used in applications ranging from large-scale solar farms to small autonomous sensing devices. Research has been carried out into the integration of solar cells with the energy storage systems, and impressive achievements have been made in the integration of solar cells and batteries in a single package.^1,2^ However, this technology often needs additional electronics to match the required output of the solar cell to the input of the battery or capacitor, which increases ohmic contact losses as well as adds to the device complexity.^3^ These issues can be overcome by developing advanced photoelectrodes, which have the combined characteristics of solar energy harvesting and electrochemical energy storage in the same material. Researchers have recently developed a few photo-rechargeable energy storage systems including redox flow batteries, light-assisted lithium-ion batteries (LIBs), lithium–air batteries, dye-sensitized batteries, and electrochemical capacitors.^4−11^ Some of them can be recharged by light without the need of external electrical grids or solar calls. While these systems are promising, however, some of them frequently suffer from poor cycling stabilities, low capacities, as well as very limited photon to charge storage conversion efficiency. To try and address some of these challenges, our research group recently developed photo-rechargeable zinc-ion batteries (*h*ν-ZIBs).^12,13^ One particular advantage of ZIBs is the relatively good stability of Zn metal anodes during cycling in comparison with Li metal. Therefore, *Zn metal* can be used more readily as the anode material which simplifies the cell design.^14,15^

Previously reported *h*ν-ZIBs rely on V_2_O_5_ or VO_2_ as photoactive cathode materials, whereas in this work, we propose to use molybdenum disulfide (MoS_2_) instead.^12,13^ In addition, in previous *h*ν-ZIBs, the active material was physically mixed with charge transfer materials, binder (PVDF), and conductive additive. These random mixtures of electrode materials with conductive additives and binders can result in poor separation and transportation of photogenerated charges and limit the overall photocharge conversion efficiency. Rather than using the above drop-casting approach, we report photocathodes for *h*ν-ZIB, where we start from carbon felt (CF) collector electrode on which we directly synthesize a zinc oxide film as an electron transport and hole blocking layer. Subsequently, a molybdenum disulfide (MoS_2_) layer is deposited, which allows for both generating photoexcited electron–hole pairs and simultaneously storing Zn ions. The band gap of MoS_2_ (∼1.9 eV) is lower than those of previously reported V_2_O_5_ (∼2.2 eV) and VO_2_ (∼2.3 eV) cathodes and aligns better with the solar spectrum, which is important for the device’s overall solar energy conversion efficiency. Finally, previous reports on photobatteries are often relying on toxic materials such as Pb or V_*x*_O_*y*_, whereas MoS_2_ is a benign material.^3,12,13,16^ We found that these photocathodes can be recharged by light without any external circuit, and their capacity is enhanced by exposure to light (e.g., 245 to 340 mA h g^–1^ at a specific current of 100 mA g^–1^ and a light power of 12 mW cm^–2^ at 455 nm). The proposed binder-free and layer-by-layer coated photocathodes offer higher photoconversion efficiencies of ∼1.8% (∼0.2% of solar-conversion efficiencies) than previous *h*ν-ZIBs (0.18–1.2%) as well as shorter charging times.^12,13^

## Results and Discussion

**Figure 1:**
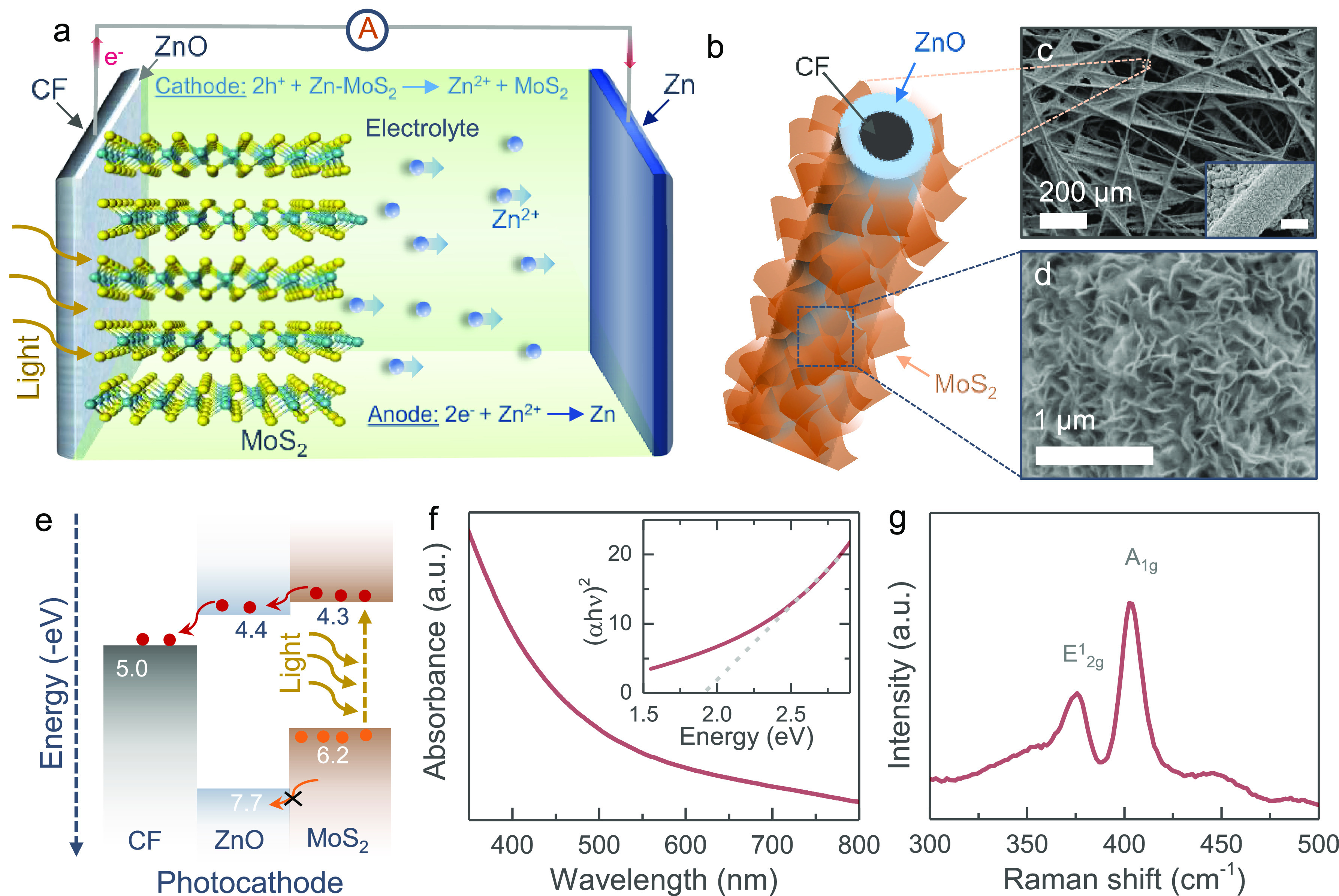
(a) Schematic illustration of the proposed photocharging mechanism of *h*ν-ZIBs. (b) Schematic illustration of MoS_2_ nanosheets grown on a ZnO coated carbon fiber. (c, d) SEM images of the photocathode at low and high magnifications. Scale ∼5 μm in the inset of part c. (e) Energy band diagram of the MoS_2_/ZnO photocathode. (f) UV–vis absorption spectrum and Tauc plot of the as-grown 2D MoS_2_ nanosheets. (g) Raman spectrum of the photocathode.

Figure 1a depicts the photocharging mechanism of a *h*ν-ZIB composed of a MoS_2_/ZnO photocathode, where the photocathodes are designed to separate and transport the photogenerated charges required to achieve photocharging (see further). These photocathodes are obtained by coating a thin layer of ZnO on a CF current collector, followed by subsequent hydrothermal growth of MoS_2_ nanosheets (see Figure 1b and the Methods section). Figure 1c,d shows scanning electron microscope (SEM) images of the photocathodes, demonstrating the successful growth of dense MoS_2_ nanosheets on a ZnO/CF current collector. Figure 1e illustrates the energy band diagram of the MoS_2_/ZnO photocathode. The layer-by-layer design of MoS_2_ and ZnO offers an energetically favorable pathway for the transport of the photogenerated electrons from MoS_2_ into CF through ZnO. This ZnO layer also blocks holes, and the combined action of electron extraction and trapping of holes leads to the desired photocharging of the battery (see further). The UV–vis absorption spectrum (Figure 1f) of MoS_2_ nanosheets confirms a direct band gap energy of ∼1.9 eV (see Tauc plot inset), corresponding to 2H-MoS_2_ as confirmed by the Raman spectrum in Figure 1g. SEM images and X-ray diffraction (XRD) patterns of the photocathodes are provided in the Supporting Information (see Figures S1 and S2). Further, the experimental estimations of valence band and conduction band positions of MoS_2_ and ZnO are included in the Supporting Information (Figure S3 and Table S1).

**Figure 2:**
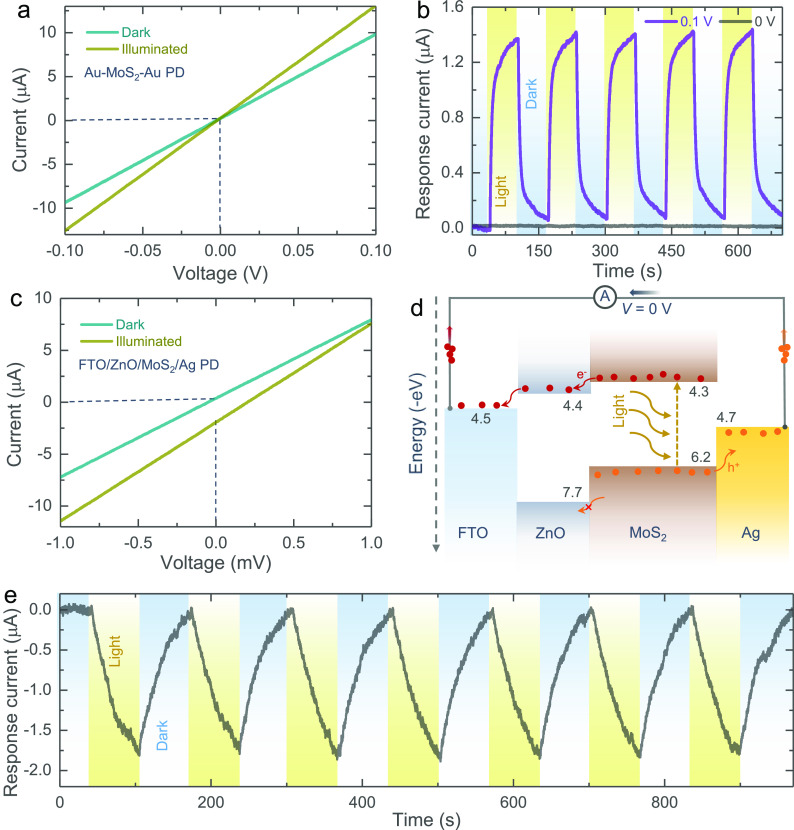
(a) *I*–*V* curves of an interdigitated Au–MoS_2_–Au PD in dark and illuminated conditions. (b) *I*–*t* tests of the Au–MoS_2_–Au PD under alternating dark and illuminated (λ ∼ 455 nm) states at *V* = 0 V and *V* = 0.1 V. (c) *I*–*V* curves of a stacked FTO/ZnO/MoS_2_/Ag PD in dark and illuminated (λ ∼ 455 nm) states. (d) Energy band diagram of the stacked FTO/ZnO/MoS_2_/Ag PD at *V* = 0 V. (e) *I*–*t* measurement of the stacked FTO/ZnO/MoS_2_/Ag PD under alternating dark and light (λ ∼ 455 nm) conditions at *V* = 0 V.

Next, to study the separation of photoexcited charges between MoS_2_ and ZnO, we have fabricated planar metal–semiconductor–metal (MSM) type and stack type photodetectors (PDs). The details of the fabrication and electrical measurements are explained in the Methods section, and pictures of the devices are provided in Figure S4. Figure 2a shows the current–voltage (*I–V*) curves in dark and illuminated (λ ∼ 455 nm) conditions for a planar MSM PD using gold (Au) interdigitated electrodes (IDEs) on which MoS_2_ nanosheets are cast. The increase in the current of the MSM PD under illumination (photocurrent) confirms the photosensitivity of the MoS_2_ nanosheets. Furthermore, the currents in dark and illuminated conditions intersect at 0 V, which suggests the need for an external driving force (i.e., bias voltage) to separate photogenerated electrons and holes in MoS_2_ under illumination. This can be further confirmed from the current–time (*I*–*t*) PD tests in alternating dark and illuminated conditions at the absence (*V* = 0 V) and presence (*V* = 0.1 V) of applied bias voltages shown in Figure 2b. To confirm the charge separation suggested in Figure 1e, we fabricated a PD consisting of a layer-by-layer stack of MoS_2_ and ZnO on fluorine doped tin oxide (FTO) transparent substrates with a silver (Ag) top contact. These devices show a photocurrent generation even in the absence of an external bias voltage (*V* = 0 V) as shown in the *I*–*V* curves (Figure 2c), as expected from the energy band diagram (Figure 2d). Therefore, the proposed MoS_2_ and ZnO material stack is capable of separating photogenerated charges under illumination and is suitable to develop photo-rechargeable batteries (see further). Figure 2e shows the *I*–*t* curve of the stacked PD under alternating dark and light conditions at *V* = 0 V.

Next, the proposed photocathodes are tested in coin cells (CR2450) with a ∼8 mm diameter glass window (see the Methods section). As mentioned above, Zn metal anodes are stable during cycling compared to Li metal, and therefore, Zn metal anodes are used as a standard anode material in this field and in this paper. Throughout this paper, we will use an aqueous 3 M Zn(CF_3_SO_3_)_2_ electrolyte (see the Methods section).

**Figure 3:**
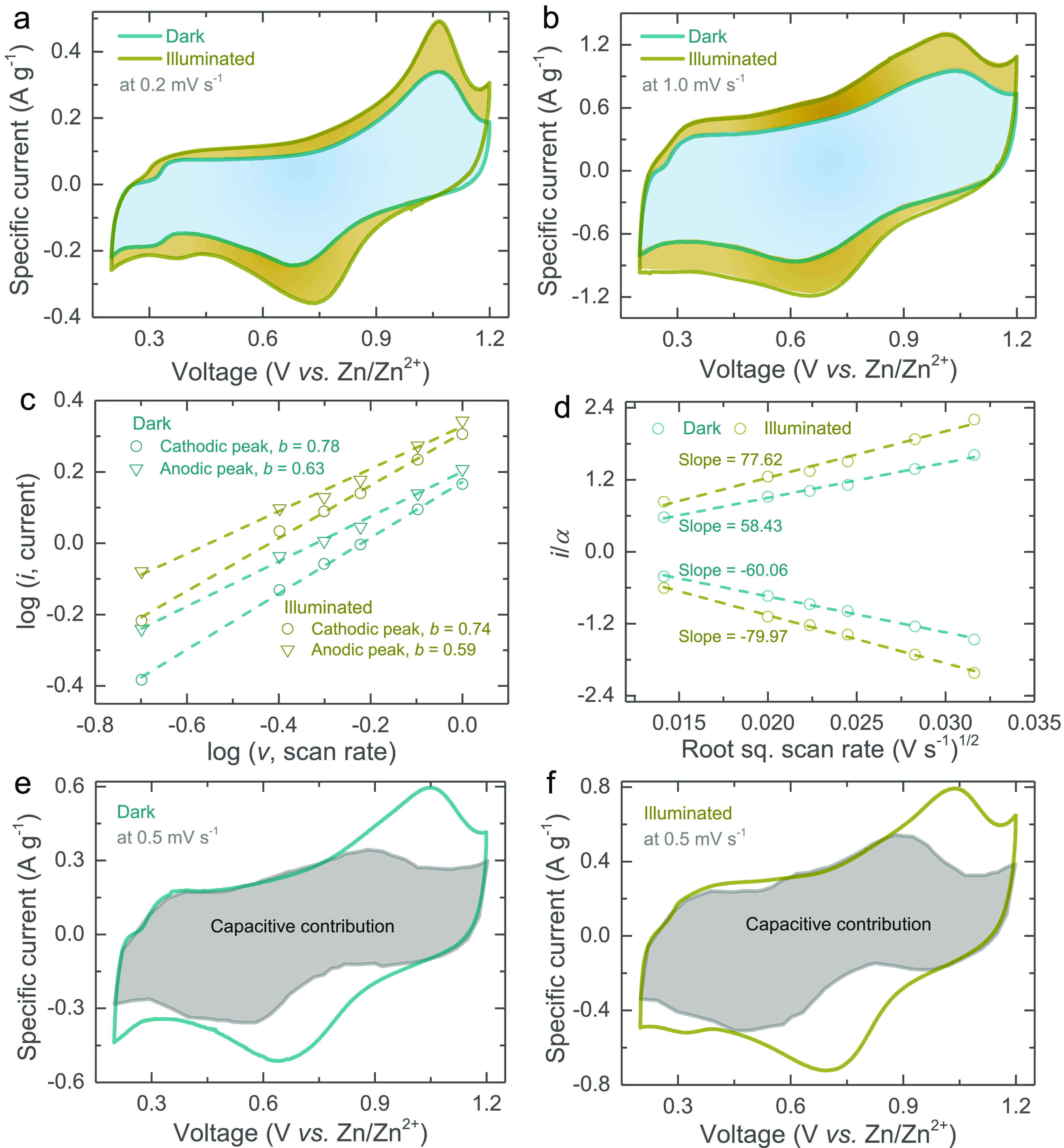
CV curves of the *h*ν-ZIBs at scan rates of (a) 0.2 mV s^–1^ and (b) 1.0 mV s^–1^ in dark and illuminated states. (c) *b*-value determination in dark and illuminated states. (d) Comparative analysis of diffusion constants in dark and illuminated conditions. (e, f) Capacitive contribution determinations to charge storage at 0.5 mV s^–1^ in dark and illuminated conditions.

To analyze the electrochemical performance of the *h*ν-ZIBs, we first carry out cyclic voltammograms (CV) at different scan rates, ranging from 0.2 to 1.0 mV s^–1^ (voltage window of 0.2–1.2 V) in dark and illuminated conditions. An LED with a 455 nm wavelength and intensity of ∼12 mW cm^–2^ is used as a light source, unless stated otherwise. As shown in Figure 3a,b, the CV responses of the *h*ν-ZIBs at scans of 0.2 and 1.0 mV s^–1^ in the dark condition show a pair of cathodic and anodic peaks that correspond to intercalation and deintercalation reactions.^17^ The peak currents for both cathodic and anodic sweeps are significantly increased under illumination because of the photosensitivity of the photocathodes. The area of the CV curves increases with approximately 39.5% at 0.2 mV s^–1^ and 40.1% at 1.0 mV s^–1^ CV when illuminated. Additional CV curves at scan rates of 0.4, 0.5, 0.6, and 0.8 mV s^–1^ in dark and illuminated conditions are provided in Figure S5 (Supporting Information). From these data, the capacitive and diffusive energy storage contributions can be analyzed by the relationship between the peak current (*i*) and scan rate (*v*) as follows
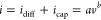
or
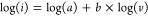
where, *i*_diff_ represents diffusion-limited current, *i*_cap_ is capacitive-limited current, and *a* and *b* are adjustable parameters. The value of *b* defines the type of electrochemical charge storage reaction; if *b* is ∼0.5, the charge storage is dominated by diffusion processes, while if *b* is ∼1, the process is capacitive.^18^ As shown in Figure 3c, the log(*i*) vs log(*v*) plots, the calculated *b*-values for the cathodic/anodic peaks are ∼0.78/∼0.63 and ∼0.74/∼0.59, respectively, in dark and illuminated conditions. These results indicate that charge storage contributions from both capacitive and diffusion-controlled processes are taking place with possibly a slight shift toward more diffusion-controlled contributions when the light is turned on. We also use the above CV data to study the diffusion constant (*D*) using the equations below
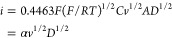
where *F* is Faraday’s constant, *C* is the initial concentration (mol cm^–3^), *A* is the photocathode area (cm^2^), and α = 0.4463*F*(*F*/*RT*)^1/2^*CA* is a constant.^19^ The area *A* is difficult to measure, but if we assume that the photocathode area is not influenced by illumination, then we can calculate the ratio of the diffusion constant in dark and light conditions. From the slopes of *v*^1/2^ vs *i*/α plots, the relative increases in the diffusion constant under illumination are ∼33.2% and ∼32.8% for cathodic and anodic peaks.

Finally, the charge storage contributions can be split into capacitive-controlled (*k*_1_*v*) and diffusion-controlled (*k*_2_*v*^*1/2*^) parts as a function of the voltage and can be expressed by^20^
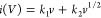
or
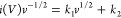


We carried out these calculations for the voltage range studies in our CV measurements, as illustrated in Figure 3e for dark conditions and Figure 3f for illuminated conditions. Overall, the capacitive contributions according to this equation are ∼72.8% and ∼71.9% in dark and light conditions, or in other words, the photogenerated charges very slightly improved the diffusive contribution of the photocathodes, which is in agreement with the results discussed above for Figure 3c.

**Figure 4:**
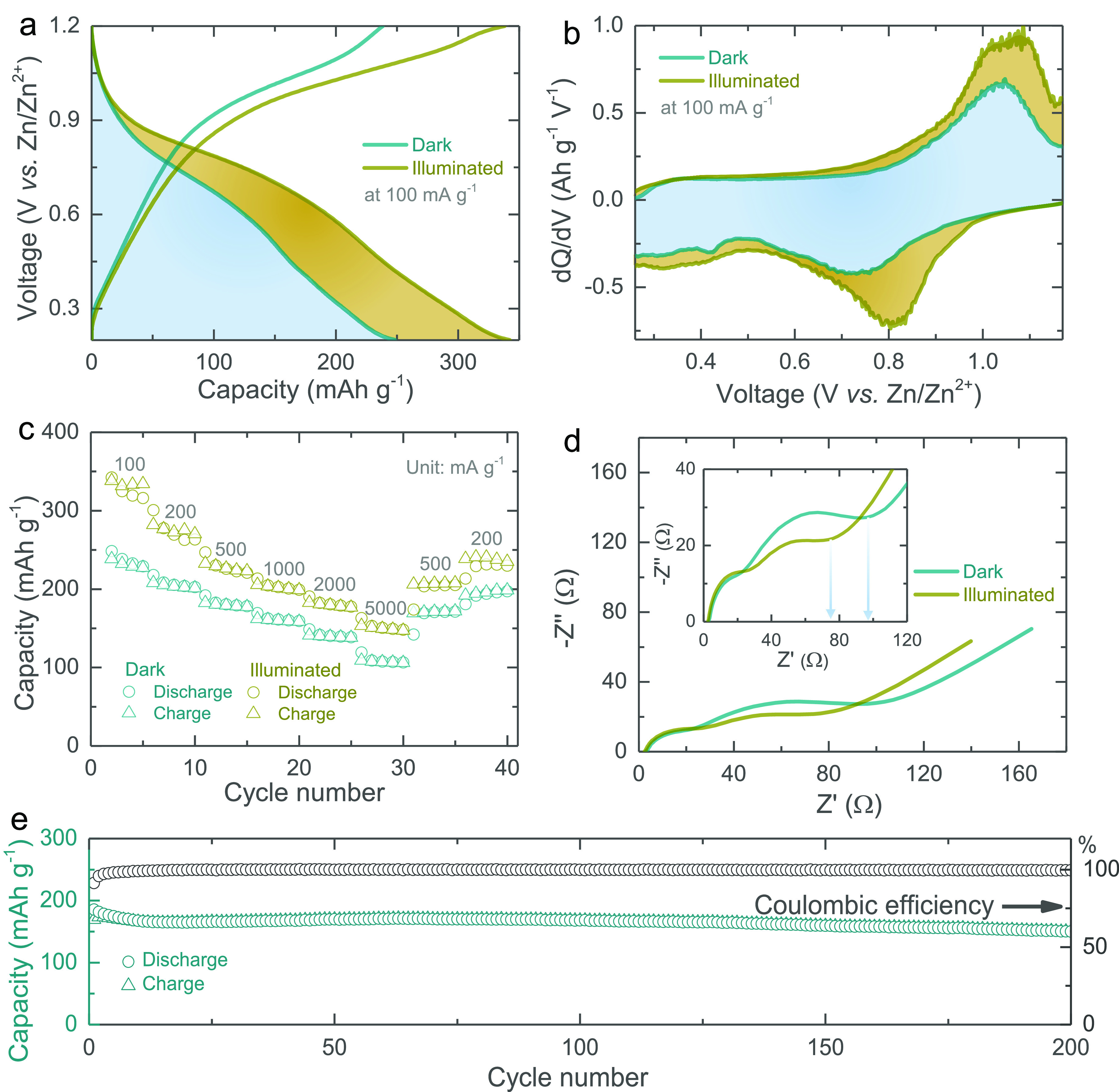
(a, b) Galvanostatic discharge–charge curves and the respective d*Q*/d*V* curves at 100 mA g^–1^ in dark and illuminated states. (c) Rate capacity tests both in dark and in illuminated states. (d) AC impedance spectra acquired in the frequency range from 10 mHz to 100 kHz at 10 mV of the *h*ν-ZIB in dark and illuminated states. (e) Specific capacity of the *h*ν-ZIB for the first 200 discharge–charge cycles tested at 500 mA g^–1^ in the dark.

Next, we measure galvanostatic discharge–charge curves at different specific currents (100–5000 mA g^–1^) in dark and illuminated conditions (cutoff voltages 0.2–1.2 V). The measured capacities increase under illumination due to photogenerated charge carriers. For instance, at a specific current of 100 mA g^–1^, the capacity increases from 245 to 340 mA h g^–1^ (38.77% enhancement) under illumination as illustrated in Figure 4a. The d*Q*/d*V* data in dark and light conditions shown in Figure 4b (100 mA g^–1^) are in agreement with the CV data reported above. Rate test results are shown in Figure 4c, and the corresponding galvanostatic discharge–charge curves at specific currents of 200, 500, 1000, 2000, and 5000 mA g^–1^ in light and dark conditions are included in Figure S6. These results indicate that light can be used not only to recharge our *h*ν-ZIBs (see further) but also to increase rate performance. The electrical impedance spectroscopy (EIS) spectra in dark and light conditions (see Figure 4d) show two semicircles; we refer to previous work for more details on the EIS spectra of ZIBs.^21^ Under illumination, the charge transfer resistance decreases from ∼95 to ∼74 Ω (inset), which is in agreement with previous reports on light interactions with Zn-ion batteries.^12,13^ Finally, our *h*ν-ZIBs show a ∼82% capacity retention after 200 cycles (see Figure 4e) and 54% after 1000 cycles (see Figure S7), whereas without the ZnO coating (MoS_2_/CF), the electrode shows the capacity retention of ∼84% after 200 cycles (see Figure S8). In other words, the ZnO coating leads to a 2% capacity loss over 200 cycles, which is probably within the measurement error. Moreover, the proposed electrodes where ZnO and MoS_2_ are directly grown on CF show a better capacity retention compared to the classic slurry cast MoS_2_–SuperP–PVDF electrodes, which achieved a capacity retention of ∼80% after 200 cycles (see Figure S9). Further, a capacity retention of ∼69% was observed after 200 cycles under illumination (Figure S10). No significant changes in the photocathode morphology are noticed after 200 cycles both in dark (Figure S11a) and in illuminated (Figure S11b) conditions. However, few cracks appeared when the cycle number increased to 500 (Figure S11c,d). Further, the post-mortem SEM images of the Zn anode before and after cycling (Figure S12) show an increase in surface roughness due to zinc dendrite formation as observed in earlier reports.^22^

**Figure 5:**
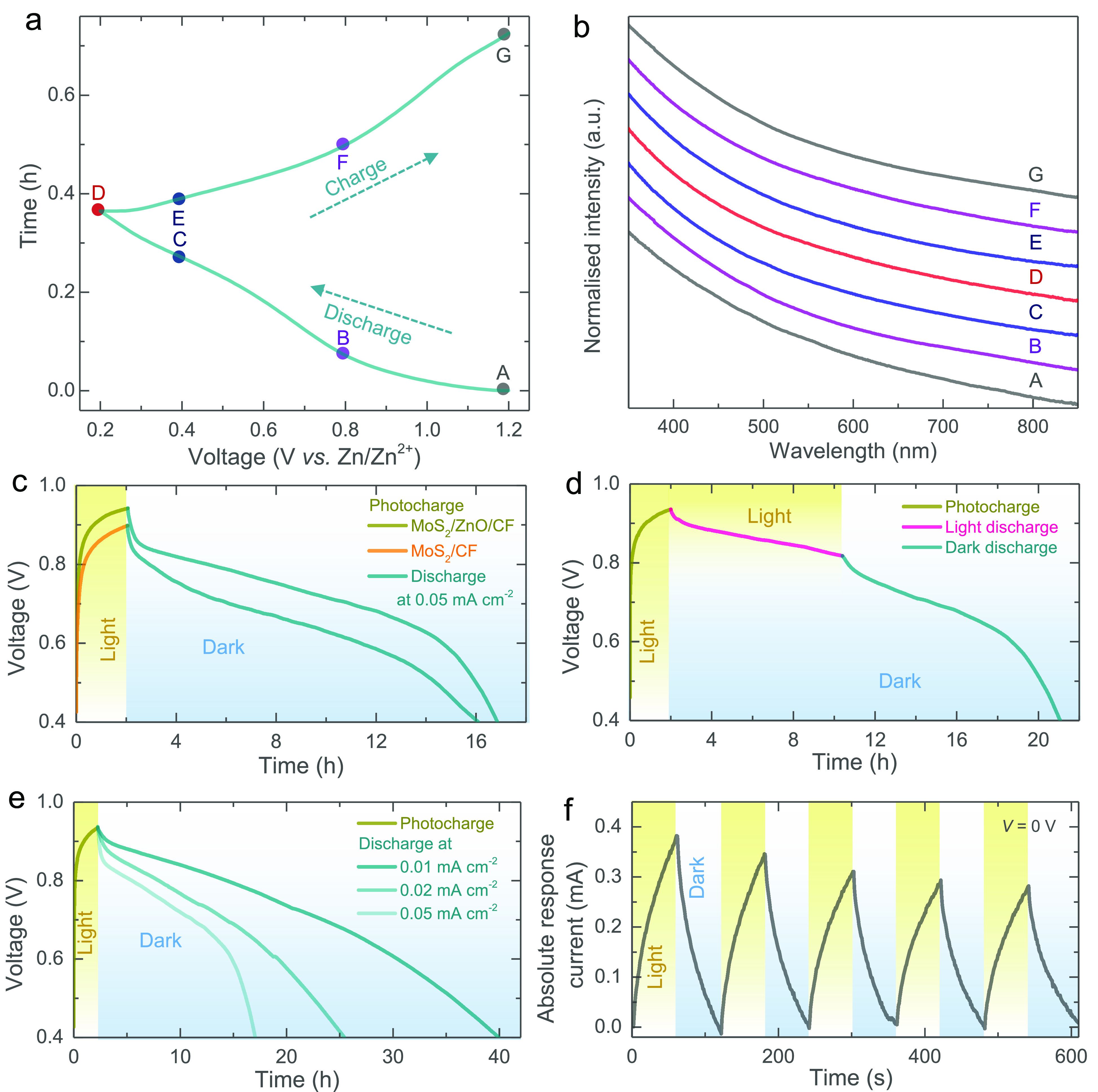
(a) Galvanostatic 2nd discharge–charge cycle of a *h*ν-ZIB. (b) Absorbance spectra of the photocathode material at different SOC indicated in part a. (c) Photocharging experiments using an LED source (λ ∼ 455 nm, 12 mW cm^–2^) of electrodes with and without the ZnO coating, followed by discharge curves at a specific current of 0.05 mA cm^–2^ in dark conditions. (d) Photocharge (λ ∼ 455 nm) followed by discharge at 0.05 mA cm^–2^ in illuminated and dark conditions. (e) Photocharge (λ ∼ 455 nm) and discharges at different specific currents. (f) Chronoamperometry test of the *h*ν-ZIB under alternating dark and illuminated (λ ∼ 455 nm) states at *V* = 0 V.

*Ex situ* UV–vis and Raman data of the photocathodes are collected to study changes in the electrode’s band gap and structural evolution as a function of the state of charge (SOC, see Figure 5a). Figure 5b shows UV–vis absorbance curves of our photocathodes at varying SOC, which suggests that the insertion of Zn ions into MoS_2_ does not notably change the band gap (Table S2 shows the band gaps of the MoS_2_ at the respective SOC). This measurement is in agreement with the above CV and galvanostatic data, which show enhancements in currents and capacities throughout the studied SOC range. Previous studies of MoS_2_ cathodes in ZIBs have shown a phase change from 2H into 1T when cycling in the voltage range 0.2–1.4 V.^17^ However, in our voltage range 0.2–1.2 V, *ex situ* Raman spectra (Figure S13a) and XRD patterns (Figure S13b) show that MoS_2_ maintains its semiconducting 2H phase which is key to the photocharging process.

Finally, Figure 5c–f shows the ability to charge our *h*ν-ZIBs with light only (no external electric power supplied). For these experiments, we discharge the battery with a constant specific current (see Figure S14) and then photocharge the battery using light only without applying an external current. After 2 h of light charging, our batteries reach a voltage of ∼0.94 V as illuminated in Figure 5c. The voltage can be increased to 0.99 V by prolonging illumination (see Figure S15a). In reference electrodes without a ZnO charge transport layer, the photocathodes only reach a voltage of 0.90 V as shown in Figure 5c. As expected from the band energies in Figure 1e, photogenerated electrons can be transported from MoS_2_ to CF, but ZnO provides more efficient electron transport while at the same time blocking holes, which we think reduces charge recombination and results in faster photocharging. Similarly, our *h*ν-ZIBs can be charged using a solar simulator with an intensity of 1 sun (400–1100 nm, LED Solar Simulator LSH-7320) as shown in Figure S15b. Furthermore, we can photocharge *h*ν-ZIBs while at the same time discharging them with a constant current, as illustrated in Figure 5d. We observe that light illumination slows down the voltage drop during discharging, which is probably due to simultaneous photocharging effects. If light is turned off during these experiments, as shown in Figure 5d after about 10 h, the discharge rate goes back to what we initially observed. Figure 5e shows the photocharge and discharges at different specific currents in the dark.

Overall, we think that, during photocharging, the photogenerated electrons are transported from the photocathode to the Zn anode through the external circuit, as confirmed from chronoamperometry measurements at *V* = 0 V applied voltage illustrated in Figure 5f. This graph shows an increase in the response current under illumination (*I*_p_ – *I*_d_; where *I*_d_ and *I*_p_ are the currents in dark and light conditions). We believe that the photogenerated holes help drive the deintercalation of Zn^2+^ ions from the photocathode and at the same time increase the oxidation state of molybdenum. These are in balance with the Zn^2+^ ions that are reduced to Zn metal by the photogenerated electrons transported to the anode. This combined action of electrons and holes allows for the photocharging of the *h*ν-ZIBs as shown in Figure 5c–f, with the following photocharging reactions:At the photocathode:
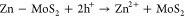
At the anode:
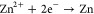


To quantify the charge stored during the photocharging process of our *h*ν-ZIBs, we calculate photoconversion efficiency (for 455 nm illumination) as well as solar-conversion efficiency (for 1 sun illumination) using the relation η = *E*_out_/*E*_in_ × 100% = *E*_out_/(*P*_in_ × *t* × *A*) × 100%, where *E*_out_ is the discharge energy, *A* is the active photocathode area, *P*_in_ represents illuminated light intensity, and *t* denotes photocharging time.^23^ The efficiencies are ∼1.8% for 455 nm illumination and ∼0.2% for 1 sun. The photoconversion efficiency of 1.8% is higher than earlier reports on photo-rechargeable ZIBs, which ranged from ∼0.18% to ∼1.2%.^12,13^ Furthermore, light charging experiments shown in Figure S16 on electrodes using the same materials but physically mixed and cast instead of the proposed layer-by-layer approach reveal a 20% lower efficiency (1.5%). The reason for these differences could be due to the improved charge transfer when MoS_2_ and ZnO are synthesized directly onto the CF electrode. Finally, the band gap of MoS_2_ (∼1.9 eV) is lower than those of V_2_O_5_ (∼2.2 eV) and VO_2_ (∼2.3 eV) and therefore better suited for solar energy harvesting, though ideally, lower band gap materials would be used. Finally, the photoconversion efficiencies of the photobatteries might be improved further through the incorporation of photoactive materials such as plasmonic nanoparticles or organic materials with photocathodes to allow efficient light harvesting, separation, and transportation of photocharge kinetics.^24−26^

## Conclusions

In summary, this work shows that MoS_2_ can be used as a photoactive cathode material for Zn-ion batteries. Compared to previous designs, the proposed electrode is using a stacked electrode architecture where ZnO is first synthesized directly on a CF collector as an electron transport and hole blocking layer, followed by MoS_2_ which acts both as the photoactive material to drive the charging process and the material storing the Zn ions. These binder-free batteries achieved a capacity of 245 mA h g^–1^ and a capacity retention of 82% over 200 cycles. The photocathodes achieved photocharge conversion efficiencies of ∼1.8%, and we observed capacity enhancements of up to 38.8% under illumination.

## Methods

### Photocathode Preparation and Characterization

First, 100 mg of zinc acetate dehydrate (Sigma-Aldrich) was dissolved in 5 mL of *N*,*N*-dimethylformamide with stirring, and then, a ZnO layer was coated on CF (Sigracet GDL 39 AA carbon graphite paper, SGL Carbon) current collectors followed by dip coating and subsequent drying at 120 °C. This process was repeated four times to obtain a uniform coating, which was finally dried at 320 °C in air. The CF current collectors were first UV/ozone treated for 1 h before coating the ZnO seed layer.

Then, ZnO coated CF substrates were transferred into a MoS_2_ growth solution of 0.076 g of ammonium molybdate tetrahydrate (Sigma-Aldrich) and 1 g of thiourea (Sigma-Aldrich) in 30 mL of deionized water; subsequently, the growth solutions with substrates were transferred into an autoclave reactor, and then, the temperature was maintained at 180 °C for 15 h. Finally, the substrates were cleaned with ethanol and deionized water and afterward dried at 70 °C. As-prepared samples were cut into specific dimensions and were used directly for electrochemical testing.

The morphological analysis and crystal structure of photocathodes were characterized by SEM (FEI Magellan 400L) and XRD (Bruker D8 Advance, Cu Kα radiation). Raman spectroscopy characterization was employed using a Renishaw InVia instrument. Further, a UV–vis–NIR spectrometer (Lamda 750) was used to characterize the optical properties.

### *h*ν-ZIB Design

Coin cell (CR2450) type *h*ν-ZIBs were obtained by making an 8 mm hole followed by sealing with a glass window inside the coin cell casing using EVO-STIK epoxy, which was kept for one night in a fume hood to dry the epoxy. Then, carbon nanotube strips (∼50 μm thick, Tortech Nano Fibers) along with a photocathode were placed sequentially inside the coin cell casing. The carbon nanotube strips were used for electrical connectivity in between the photocathode and coin cell casing as well as to avoid side reactions. Thereafter, *h*ν-ZIBs were obtained by using a Whatman glass microfiber filter paper separator, 200 μL of 3 M Zn(CF_3_SO_3_)_2_ (Sigma-Aldrich) aqueous electrolyte, along with a Zn (0.25 mm thick, Alfa Aesar) anode, and the cells were assembled following a standard procedure.

### Electrochemical Tests

CV and galvanostatic discharge–charge measurements of the *h*ν-ZIBs were tested at different scan rates (ranging from 0.2 to 1 mV s^–1^) and specific currents (100–5000 mA g^–1^) over the voltage window 0.2–1.2 V using a galvanostatic battery cycler (Biologic VMP-3) in dark and illuminated conditions. AC impedance (EIS) measurements were recorded after the second galvanostatic discharge cycle to 0.6 V in a frequency range (10 mHz to 100 kHz at a 10 mV amplitude) in dark and illuminated states. Moreover, the photocharging tests of the *h*ν-ZIBs were recorded by measuring the open circuit voltage response under illumination and then discharged by applying currents.

### *Ex Situ* Raman and UV–Vis Measurements

To measure the *ex situ* Raman spectra, the photocathodes were discharged and charged to the different SOC at a specific current of 500 mA g^–1^. The cycled electrodes were cleaned with deionized water followed by drying at 120 °C in a vacuum oven, and then, Raman spectra were acquired using a Renishaw InVia instrument. Likewise, the UV–vis measurements of the cycled electrodes were measured using a PerkinElmer UV/vis/NIR spectrometer (Lamda 750) followed by dispersing materials in ethanol.

### Fabrication of PDs and Electrical Measurements

The electrical photoresponses of MoS_2_ were studied by patterning Au/chromium (Cr) (40/10 nm) IDEs on a Si_3_N_4_/Si wafer using UV lithography followed by drop-casting MoS_2_ on the IDEs. First, *I*–*V* tests are recorded by sweeping the voltage from −0.1 to +0.1 V in dark and illuminated conditions. Then, *I*–*t* tests under alternating dark and illuminated conditions were measured both in the absence (*V* = 0 V) and in the presence (*V* = 0.1 V) of an external bias voltage. Moreover, the stack type FTO/ZnO/MoS_2_/Ag PD was fabricated using a layer-by-layer coating of ZnO and MoS_2_ on the FTO coated glass substrate followed by drying at 120 °C in a vacuum oven. Finally, Ag conducting paste contacts were used, and then, *I*–*V* tests were performed in the sweeping voltage from −1 to +1 mV (in dark and illuminated states) as well as *I–t* measurements in alternating dark and illuminated conditions in the absence of bias voltage (*V* = 0 V).

## Supporting Information Available

SEM images and XRD patterns; CV results; UV–vis; estimation of valence band and conduction band positions; digital images; galvanostatic discharge–charge curves; long-term cycling; SEM images; band gaps; *ex situ* Raman spectra; discharge cycle; prolong photocharging; photocharging under 1 sun; and photocharging of physically mixed MoS_2_ with the ZnO nanoparticle photocathode (PDF)
