# Supplementary material for: Molybdenum Disulfide–Zinc Oxide Photocathodes for Photo-Rechargeable Zinc-Ion Batteries
Source: ACS Nano. 2021 Oct 5;15(10):16616–24. doi: 10.1021/acsnano.1c06372 (PMC8552498; doi:10.1021/acsnano.1c06372)
Supplement: Supplementary file 1 — nn1c06372_si_001.pdf [file nn1c06372_si_001.pdf]

Supporting Information

for

Molybdenum Disulfide - Zinc Oxide Photocathodes for Photo-Rechargeable Zinc-Ion Batteries

Buddha Deka Boruah,[1,*] Bo Wen,[1,2] and Michael De Volder[1,*]

[1]Institute for Manufacturing, Department of Engineering, University of Cambridge, Cambridge CB3 0FS, UK

[2]Cambridge Graphene Centre, University of Cambridge, Cambridge CB3 0FA, UK

*Corresponding Authors: Dr. Buddha Deka Boruah, E-mail: bd411@cam.ac.uk

Prof. Michael De Volder, E-mail: mfld2@cam.ac.uk

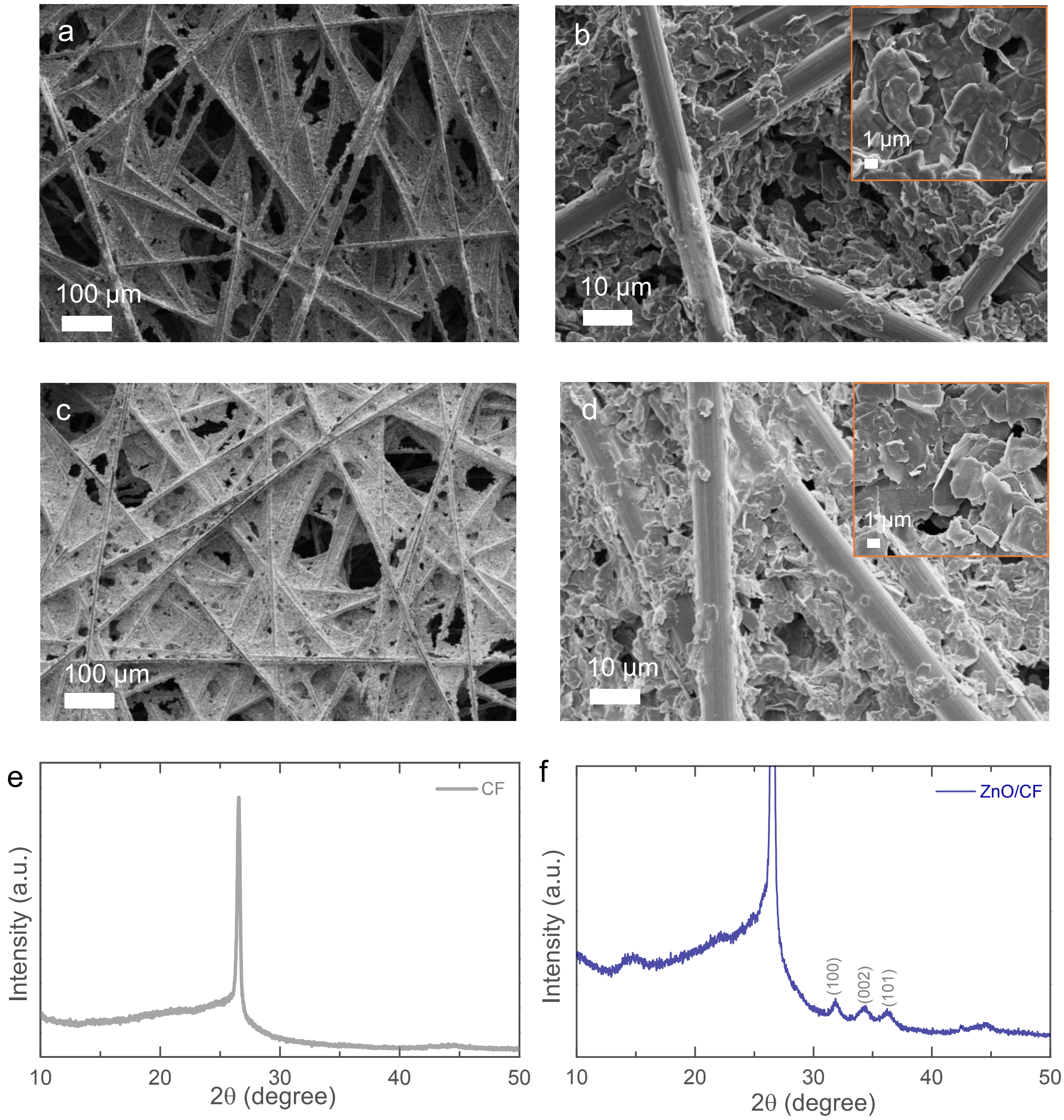

Figure S1. *SEM images of (a, b) pristine CF and (c, d) ZnO layer coated CF at low and high magnifications. (e, f) XRD patterns of the CF before and after ZnO layer coating.*

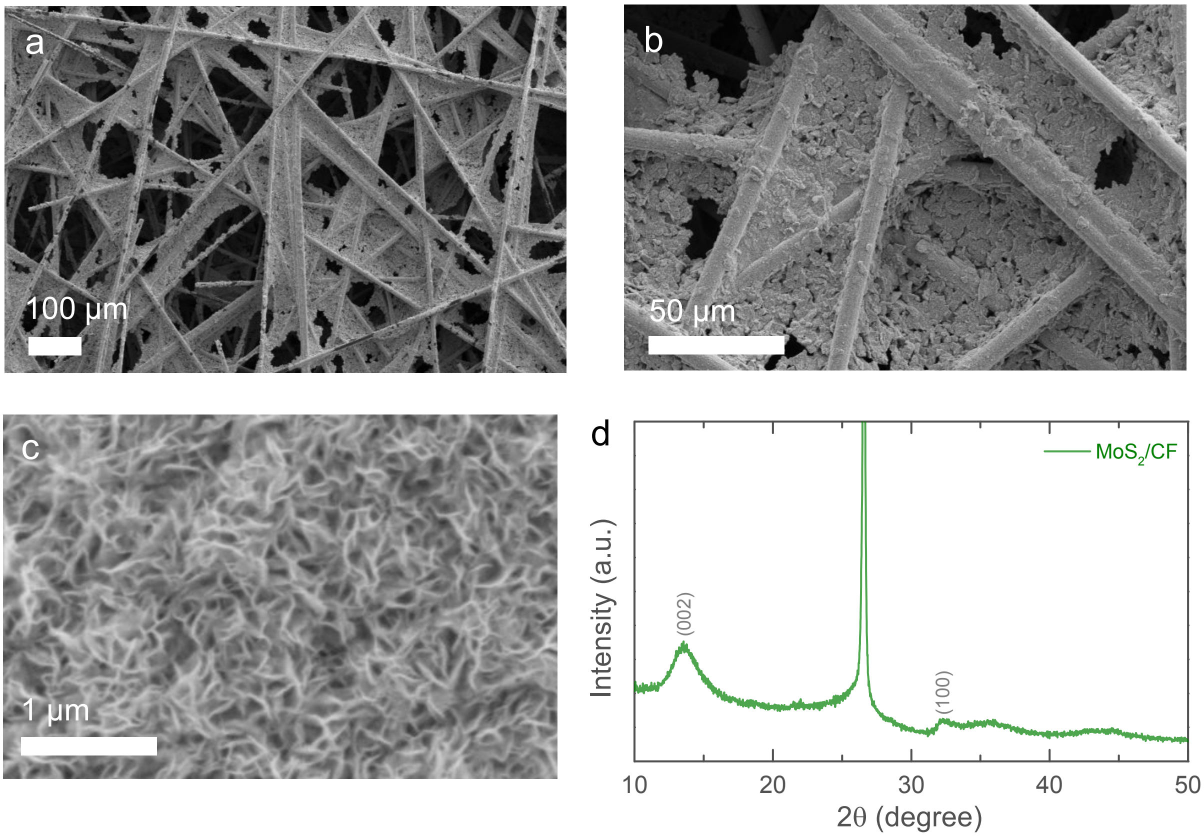

Figure S2. *(a-c) SEM images MoS_2/CF at low and high magnifications. (d) XRD pattern of the MoS_2/CF.*

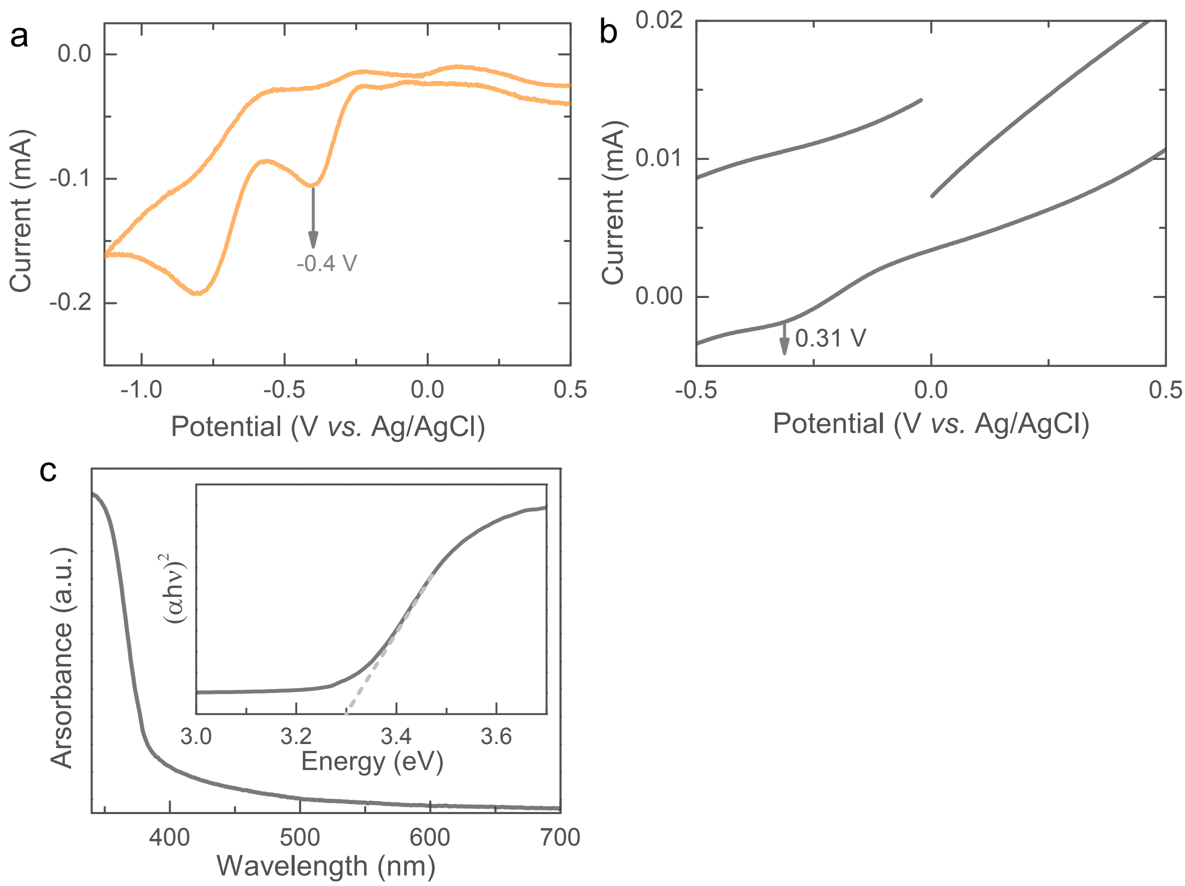

Figure S3. *CVs at a scan rate of 10 mV s^{-1} of (a) MoS_2 film on FTO coated glass substrate and (b) ZnO seed layer coted on CF, which are served as working electrodes. Pt is used as a counter electrode and Ag/AgCl is used as a reference electrode tested in 0.1 M KOH electrolyte. (c) UV-VIS absorption spectrum and Tauc plot (inset) of ZnO seed layer.*

Table S1. *Estimation of valence band and conduction band positions of MoS_2 and ZnO (E^o(Ag/AgCl) = 0.198 V).*

Material	E^o red *vs.* Ag/AgCl/KCl (V) (from CVs)	E^o red *vs.* NHE (V)	CB (eV) −(E^o *vs.* NHE + 4.5)	Optical band gap (E_g, eV)	VB (eV) −(E_g) + CB
MoS_2	-0.40	-0.20	-4.3	1.9	-6.2
ZnO	-0.31	-0.11	-4.4	3.3	-7.7

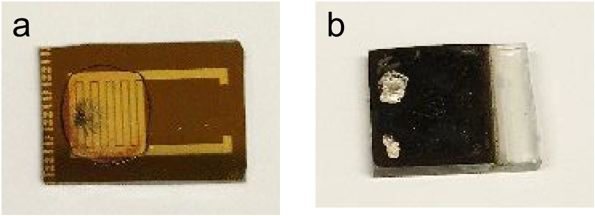

Figure S4. *Digital images of the (a) planar Au-MoS_2-Au PD and (b) stacked FTO/ZnO/MoS_2/Ag PD.*

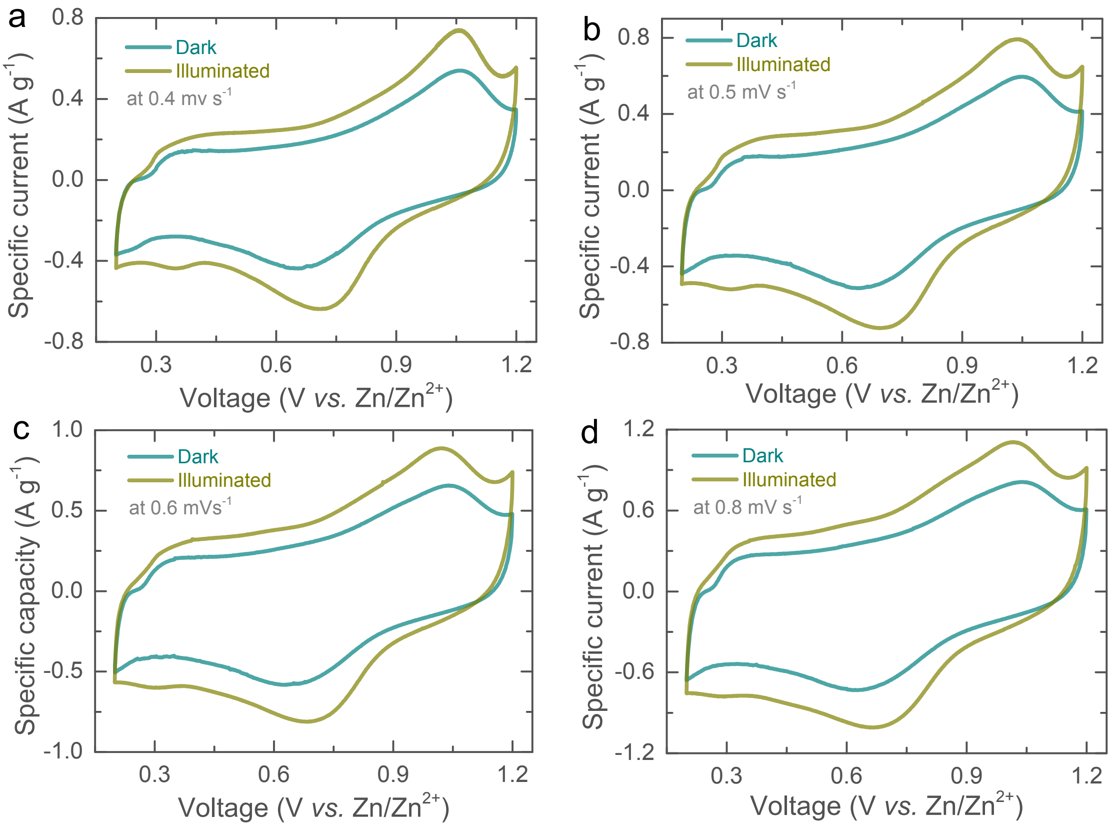

Figure S5. *CV curves of the hv-ZIBs at scan rates of (a) 0.4 mV s^{-1}, (b) 0.5 mV s^{-1}, (c) 0.6 mV s^{-1} and (d) 0.8 mV s^{-1} in dark and illuminated (λ ~ 455 nm) conditions.*

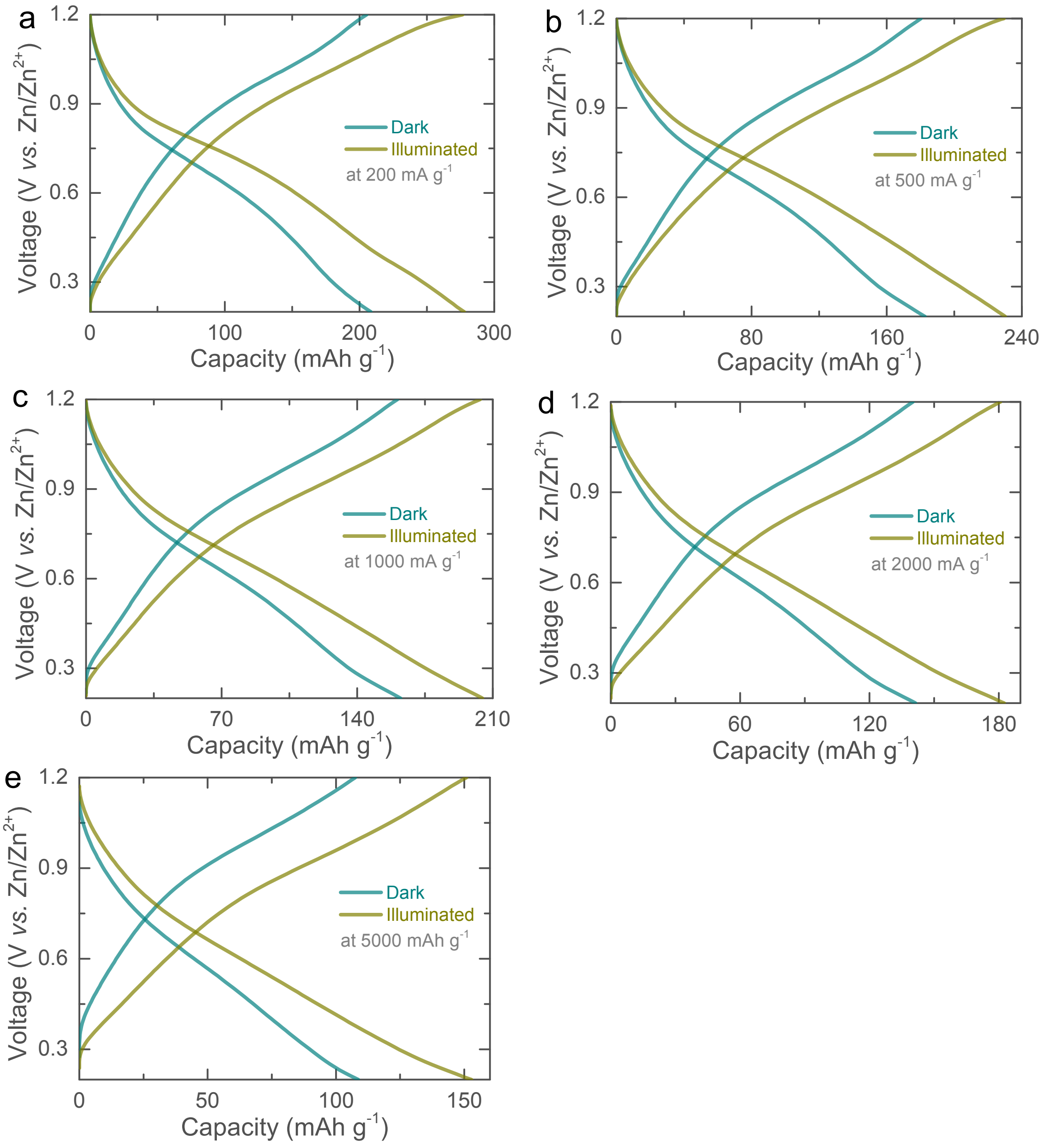

.

Figure S6. *Galvanostatic discharge-charge curves at specific currents of (a) 200 mA g^{-1}, (b) 500 mA g^{-1}, (c) 1000 mA g^{-1}, (d) 2000 mA g^{-1}, and (e) 5000 mA g^{-1} in dark and illuminated (λ ~ 455 nm) conditions.*

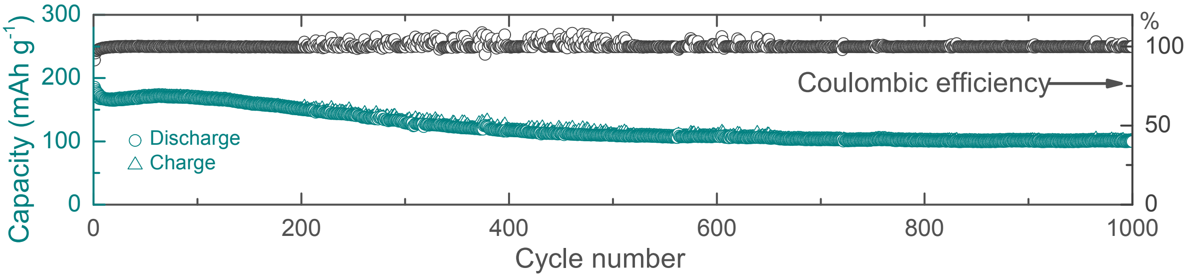

Figure S7. *Long-term cycling for 1000 cycles of the hv-ZIB at 500 mA g*$^{-1}$ *in dark.*

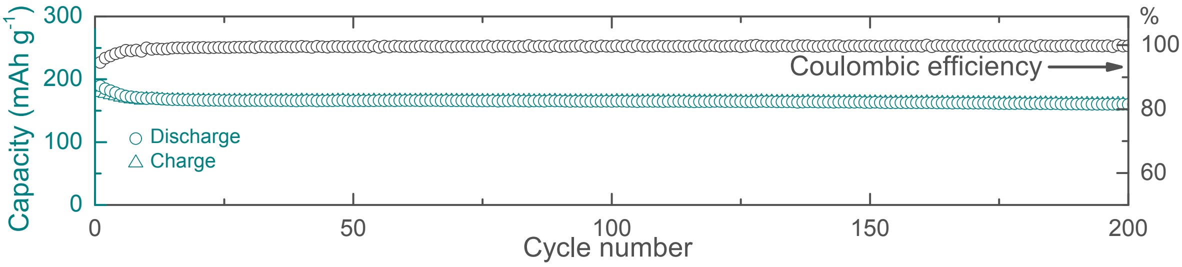

Figure S8. *Long-term cycling for 200 cycles at 500 mA g*$^{-1}$ *in dark of the without ZnO coating* MoS_2*/CF electrode based hv-ZIB.*

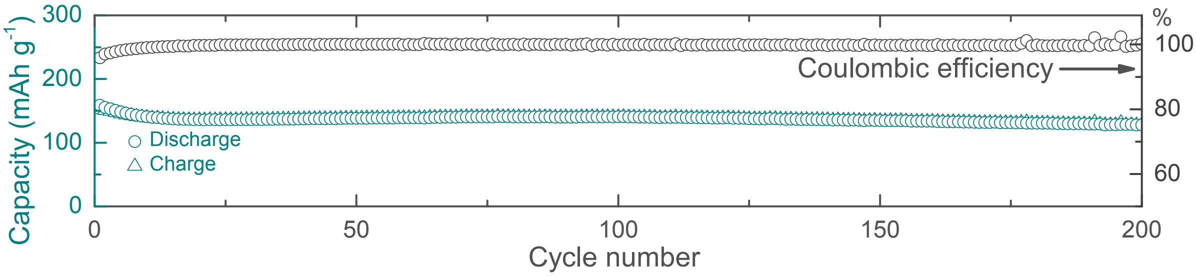

Figure S9. *Long-term cycling for 200 cycles at 500 mA g*$^{-1}$ *in dark of a classic* MoS_2*-SuperP (*MoS_2*, SuperP and PVDF in a 80:10:10 ratio) electrode based ZIB.*

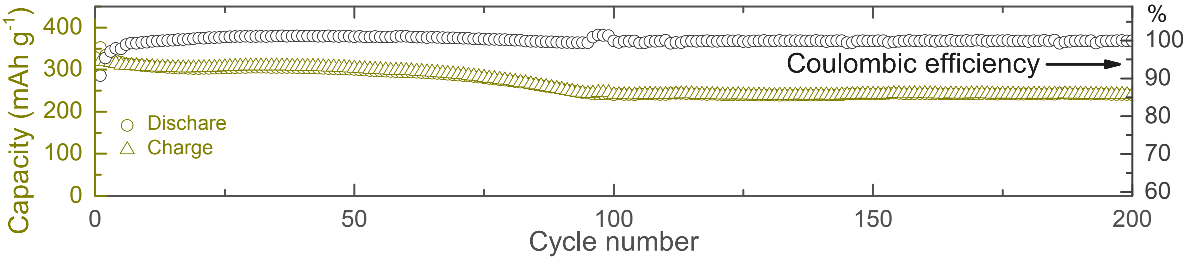

Figure S10. *Long-term cycling for 200 cycles of the hv-ZIB at 500 mA g*$^{-1}$ *under illuminated condition.*

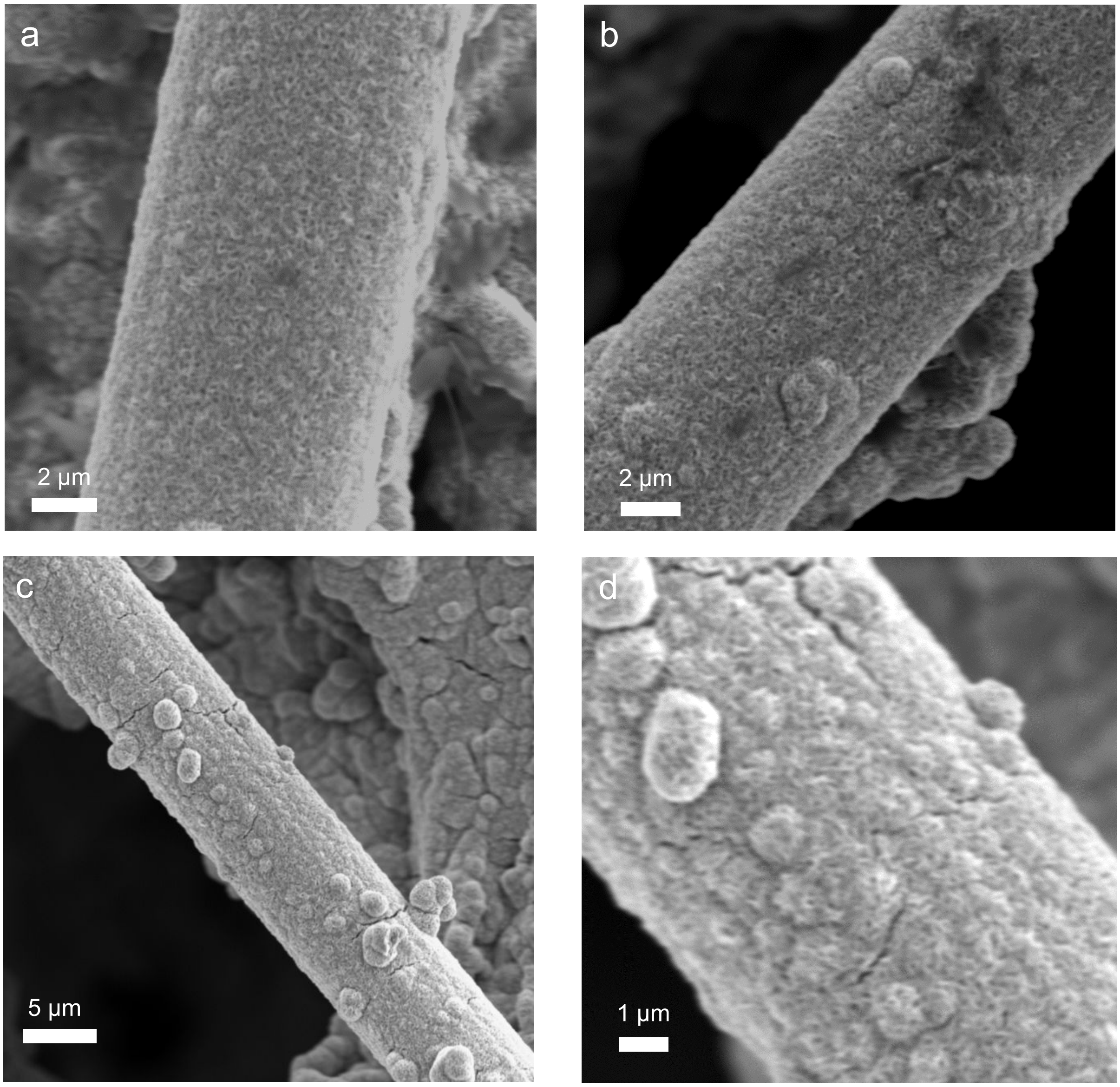

Figure S11. *(a, b) Post-mortem SEM images of cycled (200 cycles) photocathodes in dark and illuminated conditions. (c, d) Post-mortem SEM images at low and high magnifications of the cycled photocathode for 500 cycles in dark.*

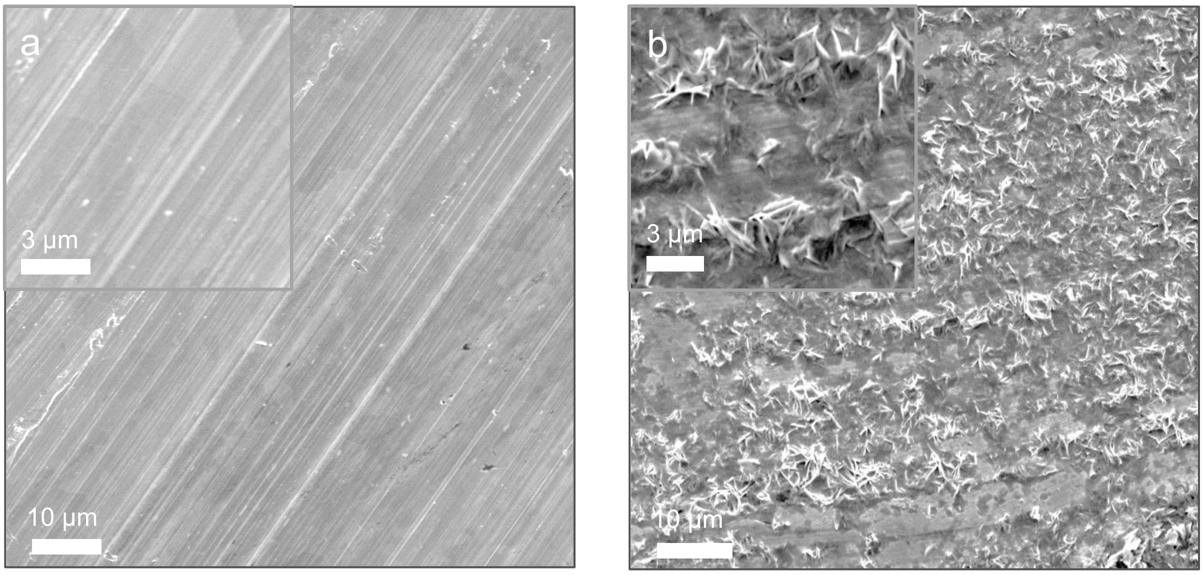

Figure S12. *(a, b) SEM images of pristine and cycled (200 cycles in dark) Zn anodes.*

Table S2. *Bandgaps of MoS_2 at different SOC.*

	**A**	**B**	**C**	**D**	**E**	**F**	**G**
Bandgap (eV)	1.93	1.96	1.94	1.95	1.95	1.94	1.94

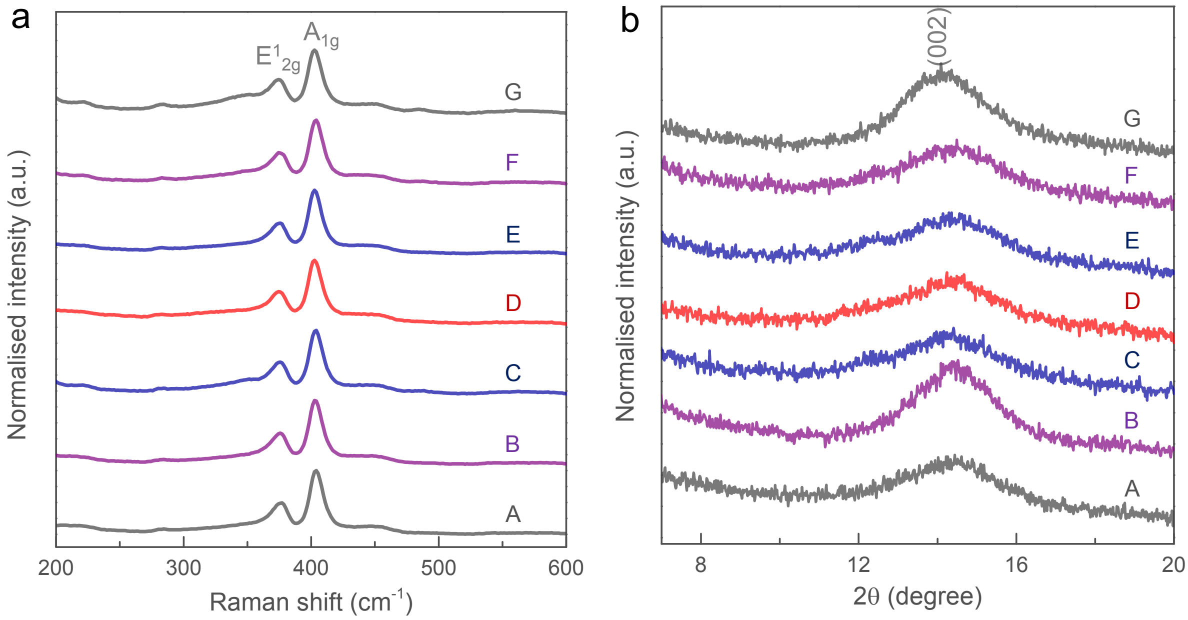

Figure S13. *(a) Raman spectra and (b) XRD patterns of the photocathodes at different SOC.*

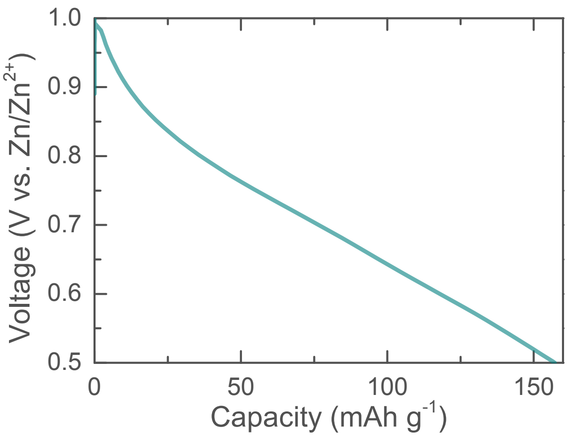

Figure S14. *Discharged cycle of the photo-charged hv-ZIB.*

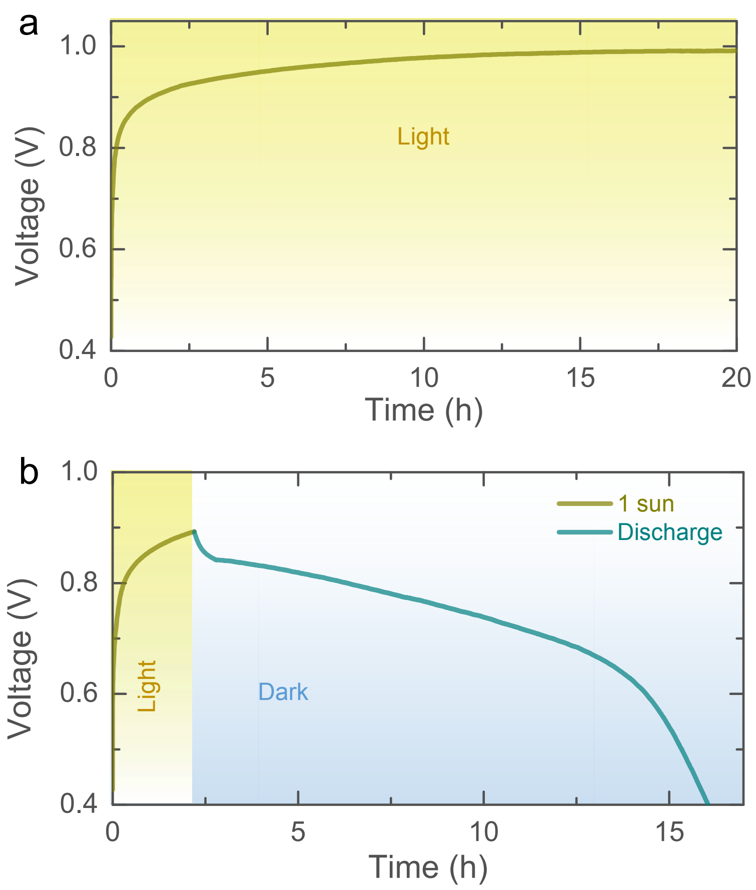

Figure S15. *(a) Prolong photocharging with 20 h of continuous light (λ ~ 455 nm) illumination. (b) Photocharge under 1 sun (400 – 1100 nm, LED Solar Simulator LSH-7320) and discharge in dark.*

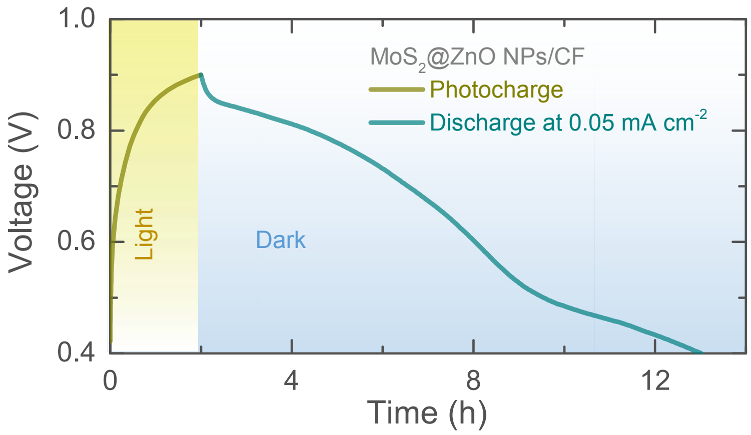

Figure S16. *Photocharge (λ ~ 455 nm) and discharge of the physically mixed MoS_2 with ZnO nanoparticles (NPs) photocathode (MoS_2, ZnO NPs and PVDF in a 93:2:5 ratio).*
